# Immune Checkpoints: Novel Therapeutic Targets to Attenuate Sepsis-Induced Immunosuppression

**DOI:** 10.3389/fimmu.2020.624272

**Published:** 2021-02-03

**Authors:** Margaret A. McBride, Tazeen K. Patil, Julia K. Bohannon, Antonio Hernandez, Edward R. Sherwood, Naeem K. Patil

**Affiliations:** ^1^Department of Pathology, Microbiology and Immunology, Vanderbilt University Medical Center, Nashville, TN, United States; ^2^Department of Anesthesiology, Vanderbilt University Medical Center, Nashville, TN, United States

**Keywords:** sepsis, immunosuppression, T lymphocyte, myeloid cell, immune checkpoints

## Abstract

Sepsis is a leading cause of death in intensive care units and survivors develop prolonged immunosuppression and a high incidence of recurrent infections. No definitive therapy exists to treat sepsis and physicians rely on supportive care including antibiotics, intravenous fluids, and vasopressors. With the rising incidence of antibiotic resistant microbes, it is becoming increasingly critical to discover novel therapeutics. Sepsis-induced leukocyte dysfunction and immunosuppression is recognized as an important contributor towards increased morbidity and mortality. Pre-clinical and clinical studies show that specific cell surface inhibitory immune checkpoint receptors and ligands including PD-1, PD-L1, CTLA4, BTLA, TIM3, OX40, and 2B4 play important roles in the pathophysiology of sepsis by mediating a fine balance between host immune competency and immunosuppression. Pre-clinical studies targeting the inhibitory effects of these immune checkpoints have demonstrated reversal of leukocyte dysfunction and improved host resistance of infection. Measurement of immune checkpoint expression on peripheral blood leukocytes may serve as a means of stratifying patients to direct individualized therapy. This review focuses on advances in our understanding of the role of immune checkpoints in the host response to infections, and the potential clinical application of therapeutics targeting the inhibitory immune checkpoint pathways for the management of septic patients.

## Introduction

Sepsis is defined as life-threatening organ dysfunction caused by a dysregulated host response to infection (Sepsis-3 guidelines) (1), and accounts for an annual worldwide case load of 48.9 million patients, leading to 11 million deaths (2). In the United States alone, sepsis is among the costliest hospital conditions and accounts for an annual healthcare burden of ~$24 billion (3). Although recent advances in fluid resuscitation, antibiotic administration, and organ support have decreased in-hospital deaths, septic patients manifest increased long-term morbidity and mortality. Many sepsis survivors suffer from long-term physical and cognitive disabilities and have a higher mortality rate than the general population (4–8). Specifically, sepsis survivors suffer a death rate of 15% in the first year after hospital discharge and a rate of 6–8% per year for the next 5 years.

Sepsis is historically considered a highly inflammatory disease, however clinical trials targeting inflammation have failed and no FDA approved therapy currently exists to treat sepsis (9). Therefore, there is a critical need to advance our understanding of sepsis pathology and identify novel biological targets for the development of new therapeutics. Numerous studies show that sepsis is not only characterized by early severe inflammation but also a concurrent immunosuppressed state that may persist for months after the initial episode of sepsis (10). Importantly, the persistent inflammatory and immunosuppressed states caused by dysfunctional innate and adaptive immune responses drive impaired immunity, multi-organ injury, prolonged hospital length of stay, and death (10–13). Sepsis survivors develop an impaired ability to clear primary infections and manifest an increased incidence of secondary nosocomial infections caused by opportunistic pathogens, indicating significant immunosuppression (14, 15). Studies demonstrate that sepsis causes significant depletion of CD4 and CD8 T cells, which is a major driver of impaired immune responses and reduced host antimicrobial capacity (16–18). Leukocytes harbor specific cell surface checkpoint proteins, which are meant to limit over activation and maintain homeostasis. Immune checkpoint receptors are specific membrane molecules located predominantly, but not exclusively, on T lymphocytes, which recognize complimentary ligands on antigen presenting cells (APCs) such as monocytes, macrophages, and dendritic cells. Major cell surface inhibitory immune checkpoints include programmed cell death receptor-1 (PD-1), programmed cell death receptor ligand-1 (PD-L1), programmed cell death receptor ligand-2 (PD-L2), cytotoxic T lymphocyte antigen-4 (CTLA-4), B and T lymphocyte attenuator (BTLA), lymphocyte activation-gene-3 (LAG-3) and T cell membrane protein-3 (TIM-3) and 2B4. Expression of these inhibitory checkpoint receptors is increased during sepsis, and hypothesized to facilitate sepsis-induced immunosuppression by impairing leukocyte antimicrobial functions (19, 20) (summarized in **Table 1**). Therapeutics targeting checkpoint inhibitors in preclinical studies and early clinical trials caused improved host resistance to infections and outcomes (18, 19, 25, 62). Certain cell surface immune checkpoints also act to drive co-stimulatory signals such as the interaction between Ox40 receptor on T cells and its cognate ligand Ox40L on APCs, and stimulation of this pathway has recently been shown to improve outcomes in a murine model of sepsis (61). This review will highlight some of the recent advances in our understanding of the role of immune checkpoints during sepsis and novel metabolism focused mechanistic aspects to be considered for their translational application in combination with other immunotherapeutics.

**Table 1:** Summary of immune checkpoints cellular distribution and function.

Immune Checkpoint Receptor	Cells Expressing Receptor	Ligand	Cells Expressing Ligand	Signaling Outcome in Sepsis
PD-1 (CD279)	- T cells - B cells - Monocytes - NK cells - Dendritic cells (21)	PD-L1	- T cells - B cells - Dendritic cells - Macrophages - Tumor cells - Lung, liver, heart, placenta, kidney and pancreas and other parenchymal cells (21, 22)	- T cell exhaustion - Decreases myeloid cell function - Intestinal injury - Lung injury (18, 23–29)
		PD-L2	- Dendritic cells - Monocytes - Lung, liver, colon, small intestine, and placenta parenchymal cells (22, 30)	- Deletion of PD-L2 improves bacterial clearance, with no observed survival benefit (30)
2B4 (CD244)	- NK cells - T cells (31)	CD48	- All hematopoietic cells in humans (32)	- T cell apoptosis - Increases PD-1 expression (33–35).
CTLA-4 (CD152)	- Activated CD4 and CD8 T cells	- CD80 - CD86	- Antigen presenting cells (36)	- T cell apoptosis - Increased mortality (37, 38)
HVEM (CD270, TNFRSF14)	- Monocytes - Dendritic cells - Neutrophils - NK cells - Resting T cells - Immature B cells (39, 40)	-BTLA (CD272)	- T cells - Monocytes - Macrophages - Dendritic cells (39–42)	- Promotes CD4 T cell death - Inhibits innate leukocyte activation - Reduces bacterial clearance - Promotes organ injury (43)
LAG-3 (CD223)	- CD4, CD8, and regulatory T cells - B cells - Dendritic cells	MHC II	- Antigen presenting cells	- Inhibits T cell responses - Decreases bacterial clearance (44)
TIM-3	- NK cells - Macrophages - Dendritic cells - Mast cells - CD4 and CD8 T cells	CEACAM1	- Microvascular endothelia - Epithelial cells - T cells - B cells - Macrophages - Dendritic cells - Tumor cells (45)	- Decreases inflammation - Decreases T cell apoptosis (46, 47)
		Galectin 9	- APCs, T cells, B cells -Liver - Small intestine - Thymus - Kidney - Spleen - Lung - Cardiac and skeletal muscle (48, 49)	
Ox40 (CD134)	- Activated CD4, CD8, and regulatory T cells - Activated NK cells (50–52)	Ox40L (CD134L, CD252, glycoprotein 34)	- Macrophages - Dendritic cells - Langerhans cells - Vascular endothelial cells - Bronchial smooth muscle cells - Mast cells - Activated NK cells (53–60)	- Improves outcomes in murine sepsis (61)

## Ox40 and Ox40L

Along with T-cell receptor ligation, optimal T cell activation requires potent costimulatory signals such as the classical interaction between CD28 on T cells and CD80/86 on APCs (63). Interaction between T cell-expressed Ox40 and Ox40L on APCs is also recognized as a potent costimulatory signal for effector T cells (64). Ox40 (CD134) is a member of the tumor necrosis factor receptor superfamily and it is primarily expressed on activated CD4 and CD8 T lymphocytes (50). Ox40 receptor consists of a single transmembrane domain and a cysteine rich extracellular domain (65). Ox40 is also expressed on regulatory T cells and activated NKT cells (51, 52). The cognate ligand of Ox40 is Ox40L, which is also named as CD134L, CD252 or glycoprotein 34 (gp34) (66). Ox40L was first identified on human T-cell leukemia virus type I (HTLV-I) transformed T cells (53). Expression of Ox40L is not only restricted to classical APCs such as macrophages (54), dendritic cells (55), and Langerhans cells (56), but is also expressed on vascular endothelial cells (57), bronchial smooth muscle cells (58), mast cells (59), and activated natural killer (NK) cells (60).

**Figure 1:**
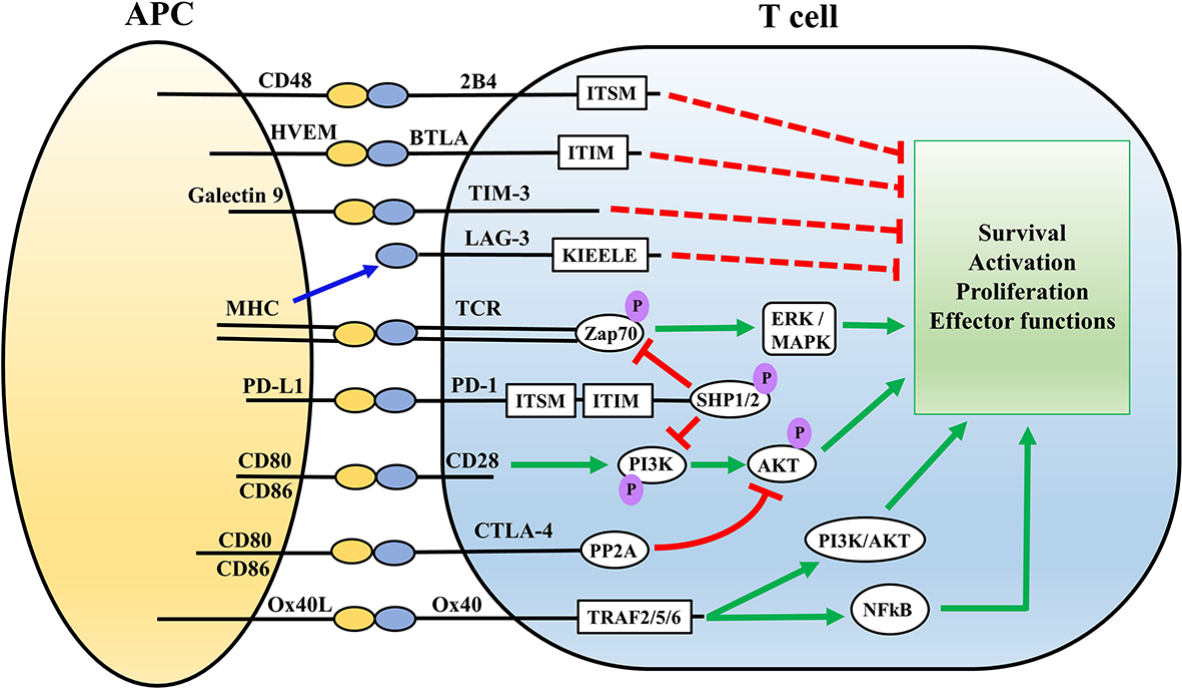
Overview of the major immune cell checkpoints and associated signaling pathways. Antigen presentation *via* MHC on APCs to the TCR complex on T cells executes activation of T cells *via* Zap70 and ERK/MAPK signaling pathways. Ligation of CD28 on T cells with CD80/86 on APCs provides co-stimulatory signals. PD-1/PD-L and CTLA-4 signaling impair T cell activation *via* inhibition of the AKT signaling pathway. PD-1signaling involves SHP mediated inhibition of Zap20 and PI3K/AKT signaling pathway, whereas CTLA-4 directly inhibits the AKT pathway *via* recruiting PP2A. 2B4, TIM3, BTLA, and LAG-3 also inhibit T cell activation through interaction with their associated ligands on APCs. Ox40-Ox40L serves as a co-stimulatory signal for T cells *via* PI3K/AKT and NFkB pathways. Red lines represent inhibitory signals while green represents stimulatory signals. ITSM, ITIM, KIEELE represent specific intracellular domains of the immune checkpoints which mediate their intracellular signaling APC, antigen presenting cell; HVEM, herpes virus entry mediator; MHC, Major histocompatibility complex; BTLA, B and T lymphocyte attenuator; TIM3, T cell membrane protein-3; LAG-3, Lymphocyte activation-gene-3; TCR, T cell receptor; PD-1, Programmed death-1; CTLA4, Cytotoxic T lymphocyte antigen-4; ITSM, Immunoreceptor Tyrosine-based Motif; ZAP70, Zeta Chain of T Cell Receptor Associated Protein Kinase 70; PI3K, Phosphoinositide 3 kinase; ITIM, immunoreceptor tyrosine-based inhibition motif; SHP, Src homology region 2 domain-containing phosphatase; PP2A, protein phosphatase 2A; TRAF, TNF receptor associated factor; ERK, Extracellular signal-regulated kinase; MAPK, mitogen activated protein kinase; AKT, protein kinase B.

Ox40 is exclusively expressed on activated CD4 and CD8 T lymphocytes and not on naïve T cells, thereby implicating Ox40-Ox40L interaction to specifically provide costimulatory signals in activated effector T cells (67). Ox40 signals *via* its cytoplasmic domain which is linked to the signal transduction pathway involving TNFR- associated factors such as TRAF2 and TRAF5 leading to NF-κB activation (**Figure 1**) (68, 69). Signaling through Ox40 drives clonal expansion of T cells, and also increases the expression of anti-apoptotic proteins such as Bcl-2 and Bcl-xl leading to increased survival of T cells (70, 71). Sepsis functionally impairs and depletes T cells (16–18). Therefore, therapeutics targeting Ox40 pathway hold significant potential to boost T cell function during sepsis.

Currently, there is great interest in the immunotherapeutic potential of Ox40 as an anticancer therapy (72), however, studies of Ox40 in sepsis are limited. A recent study by Unsinger et al., demonstrated that treatment with an agonistic antibody (Ab) to Ox40 improved T cell function and reduced mortality in a cecal ligation and puncture (CLP) model of murine sepsis (61). CLP-induced sepsis increased expression of Ox40 on splenic CD4 T cells which persisted up to 5 days, with no change on CD8 T cells. Treatment with Ox40 agonistic Ab increased splenic CD4 T cell count, and surprisingly further augmented the CLP-induced increase in splenic myeloid cell (macrophages and monocytes) numbers at day 5 post sepsis. Ox40 Ab improved T lymphocyte function, as measured by increased ability to produce IFNγ, not only in murine cells but also in peripheral blood mononuclear cells derived from sepsis patients, lending significant translational relevance to the study. It is important to note that treatment with Ox40 agonistic antibody was effective even when administered 6 and 48 h after the onset of sepsis, which closely mimics the clinical scenario for the treatment of sepsis patients. In contrast with these findings, a study by Karulf et al., showed that sepsis leads to significant upregulation of Ox40L on circulating monocytes and neutrophils at 24 h after sepsis diagnosis in their patient cohort and the level of monocyte Ox40L was higher in non-survivors (73). Treatment with a blocking antibody against Ox40L or Ox40L knock out reduced inflammation and organ damage leading to improved survival in a murine CLP model of sepsis. The protective effect of blocking Ox40L was predominantly dependent on intact macrophages and independent of T lymphocytes. A major difference between the two contrasting studies is that Unsinger et al. employed a less severe model of sepsis with 50% of septic mice surviving at day 7 post CLP, as opposed to the sepsis model by Karulf et al. which showed 100% mortality within 48 h after CLP (61, 73). Therefore, these findings indicate that there exists a fine balance for maintaining immune homeostasis during sepsis and therapeutics targeting Ox40-Ox40L could be a double-edged sword. Future studies should carefully evaluate their therapeutic potential using diverse models of sepsis in context of myeloid and T cell functions and inflammation.

## Programmed Cell Death Receptor-1 (PD-1) and PD-L1/PD-L2

PD-1 receptor, also known as CD279, is a type I transmembrane glycoprotein belonging to the immunoglobulin (Ig) superfamily and it is composed of an extracellular Variable-type (V-type) Ig domain, a transmembrane domain and an intracellular cytoplasmic signaling domain (74–76). PD-1 is expressed on variety of leukocytes including T and B lymphocytes, monocytes, NK cells, and dendritic cells (21). Signaling through PD-1 is executed upon engagement with its cognate ligands including PD-L1 and PD-L2 (77, 78). PD-L1 is expressed on activated leukocytes including T and B lymphocytes, dendritic cells, macrophages and parenchymal cells of lung, liver, heart, placenta kidney, tumor cells, and pancreas (21, 22). PD-L2 has been shown to be expressed on APCs such as dendritic cells and monocytes, along with tissues including lung, liver, colon, small intestine, and placenta (22, 30). The interaction between PD-1 and PD-L1 has been the focus of majority of the immune checkpoint studies during sepsis, while studies implicating PD-L2 are limited.

### Overview of PD-1/PD-L Signaling Pathways

The interaction between PD-1 and its ligands generates inhibitory signals to attenuate T cell responses (79). The intracellular signaling mechanisms leading to the T cell inhibitory effect of PD-1 ligation is a topic of great interest and not completely understood. The intracellular cytoplasmic domain of PD-1 is composed of tyrosine-based motifs including immuno-receptor tyrosine-based inhibitory motif (ITIM) and immuno-receptor tyrosine-based switch motif (ITSM) (75). The inhibitory effect of PD-1 proceeds predominantly though ITSM phosphorylation and to a lesser extent ITIM phosphorylation, leading to recruitment of Src homology region 2 domain-containing phosphatase-2 (SHP-2) which drives the downstream signaling pathways (75, 80). Upon PD-1 ligation, SHP-2 dephosphorylates phosphoinositide 3-kinase (PI3K) leading to inhibition of Protein Kinase B (Akt) and extracellular signal-regulated kinase/mitogen-activated protein kinase (ERK/MAPK) signaling pathways (**Figure 1**) (79, 81). SHP-2 is not the only mediator of PD-1 signaling mediated T cell exhaustion (82), and SHP-1 could compensate for absence of SHP-2 (83, 84). PD-1 mediated signaling pathway targets the primary T cell receptor (TCR) as well as the co-stimulatory CD28 pathways for downregulating T cell effector functions (85, 86). On the other hand, whether PD-L1 can reverse signal is highly debated, because the intracellular signaling domains of PD-L1 remain poorly characterized. Only a limited number of studies focused of PD-L1 harboring tumor cells have demonstrated some PD-L1 mediated effects which are independent of PD-1 (87). For example, PD-L1 has been shown to promote tumor cell proliferation and survival independent of PD-1 (88), acting through specific conserved sequence motifs to shield melanoma cells from IFNγ cytotoxicity through a STAT3/caspase-7-mediated pathway (89). Interestingly, a study by Hartley et al. showed PD-L1 delivers a constitutive inhibitory signal within macrophages, and treatment with anti-PD-L1 antibody increased proliferation, survival, and activation of murine and human macrophages which triggered tumoricidal effects of macrophages (90). Such effects, if any, for PD-L2 are unknown. The inhibitory effects of PD-1:PD-L interaction has been well documented in sepsis-induced T cell exhaustion using PD-1 and PD-L1 blocking antibodies (18, 25, 26), However, specific details about the intracellular signaling pathways executing the PD-1:PD-L interaction driven inhibitory signals are yet to be fully characterized in the context of sepsis.

### PD-1 and PD-L Mediated Immunosuppression and Organ Injury During Sepsis

Increased expression of PD-1 on T cells and PD-L1 on APCs causes T cell exhaustion during sepsis which is associated with impaired microbial clearance, multi-organ injury, and increased mortality (18, 19, 23–26). The characteristic features of T cell exhaustion include reduced co-stimulatory receptor expression such as CD28, upregulation of inhibitory immune checkpoints, and metabolic derangements leading to impaired production of effector cytokines (interferon-gamma, and granzyme-B), along with impaired proliferation and increased susceptibility to apoptotic cell death (42). A postmortem study of sepsis patients by Boomer et al. provided critical evidence that sepsis increases PD-1 and reduces CD28 and IL7 receptor expression on T cells, along with increased PD-L1 and PD-L2 expression dendritic cells (23). Increased PD-1 expression on T cells and PD-L1 expression of APCs in septic patient has been shown to be associated with lymphopenia, T cell apoptosis, and higher mortality (23, 91–93). A recent study by Wilson et al. shows upregulation of PD-1 and PD-L1 on both T and B lymphocytes, and that expression of PD-1 was found to be higher in lymphocytes associated with CD27+ memory status (94). Although, the study demonstrated an association between higher lymphocyte PD-L1 expression and secondary nosocomial infections in patients with >7 days of ICU length of stay, the expression of PD-1, PD-L1, or PD-L2 on lymphocytes was not predictive of eventual mortality (94). The findings of this study is in contrast to other studies which show an association between T cell PD-1 expression with sepsis mortality (91, 95). However, it is important to note that study conducted by Wilson et al. provided a snapshot of PD-1 expression at an early single time point within 12h of ICU admission (94), while a time course study by Tomino et al. shows that higher PD-1 expression on day 7 after ICU stay correlates with increased mortality among sepsis patients (95). Fungal *Candida* infected sepsis patients also demonstrate an increased PD-1 expression on circulating CD4 and CD8 T cells, and decreased CD28 expression (96). Studies using PD-1 knockout mice show improved survival outcomes after sepsis using a neonatal cecal slurry model (97).

In addition to T cells, PD-1/PD-L1 axis also impairs myeloid cell function during sepsis. A study by Patera et al. showed that increased PD-L1 expression on neutrophils and monocytes correlates with a decrease in their phagocytic capacity in septic patients (25). CLP-induced murine sepsis has been shown to increase PD-1 expression on liver Kuppfer cells and deletion of PD-1 improves their phagocytic capacity (98). Increased PD-L1 expression on circulating neutrophils is shown to correlate with increased pro and anti-inflammatory cytokines and higher mortality in a CLP model of sepsis (27). Shao et al. showed that increased PD-L1 expression on circulating monocytes predicts higher 28 day mortality in septic shock patients (99). Increased PD-L1 expression on NK cells within 24 h of ICU admission has recently been shown to correlate with increased Sequential Organ Failure Assessment Score (SOFA) and sepsis severity (100). From a mechanistic standpoint, a recent study by Deng et al. showed that PD-L1 expression on myeloid cells is regulated by a circadian clock gene, BMAL1 (101). Specifically, BMAL1 blocks pyruvate kinase M2 and STAT1 driven upregulation of PD-L1 expression on macrophages, reduces T cell exhaustion and immunosuppression, and protects against sepsis (101). However, detailed cellular signaling mechanisms leading to sepsis-induced upregulation of leukocyte PD-1 and PD-L1, and the downstream effects of PD-1/PD-L1 ligation needs further investigations. In the context of the ongoing COVID-19 pandemic, SARS-CoV-2 has been shown to cause lymphopenia, and impaired functional capacity of T cells and monocytes (102). However, the direct role of immune checkpoints such as PD-1/PD-L1 in mediating immunosuppression during SARS-CoV-2 infection and evaluating it as potential therapeutic target requires further investigation (103).

PD-1/PD-L signaling has also been implicated in sepsis-induced organ injury. In addition to leukocytes, PD-L1 and PD-L2 are also expressed on non-immune cells including liver, lung, kidney, small intestine, colon, tissue endothelial cells (21, 22, 30). A study by Rossi et al. showed that deletion of PD-L1 attenuated the rise in hepatic dysfunction indices including serum bilirubin, alanine aminotransferase (ALT) and aspartate amino transferase (AST) and protected the endothelial permeability barrier, which was associated with improved systemic bacteria clearance and improved survival in a murine model of CLP-induced sepsis (30). Although, deletion of PD-L2 improved systemic bacterial clearance, it did not protect against the rise in hepatic injury makers including serum bilirubin and AST, and offered no survival benefit (30). Using a CLP model of sepsis, Hutchins et al. showed increased PD-1 and PD-L1 on liver Kuppfer cells and sinusoidal endothelial cells respectively, and deletion of PD-L1 protected against sepsis-induced increase in liver vascular leakage, edema, and endothelial cell apoptosis (104). PD-L1 expression was shown to be increased in the lung tissue of postmortem samples from sepsis patients (23). Using a two hit murine model of hemorrhage followed by CLP-induced sepsis, recent studies demonstrate a deleterious role of PD-1/PD-L1 pathway in mediating sepsis-induced lung injury (29, 105, 106). Specifically, hemorrhagic shock and sepsis upregulated PD-L1 expression on lung endothelial and parenchymal cells, and deletion of PD-1 or PD-L1 attenuated sepsis-induced increase in pulmonary endothelial cell permeability and reduced lung injury (29, 106). Intravenous delivery of siRNA targeting PD-L1 expression reduces neutrophil influx into lungs and attenuates sepsis-induced lung injury, indicating targeting PD-L1 as an attractive therapeutic target to protect against sepsis-induced lung injury. Adoptive transfer of regulatory T cells (Tregs) before induction sepsis in mice subjected to hemorrhagic insult demonstrates reduced infiltration of neutrophils and lung injury, an effect which is ablated using adoptive transfer of PD-1 deleted Tregs, indicating that PD-1 expressing Tregs dampen the innate leukocyte mediated exaggerated inflammation and protect against sepsis-induced acute lung injury (105). PD-L1 also plays a critical role in regulating sepsis-induced intestinal injury. Human and murine sepsis studies show that upregulated PD-L1 expression on intestinal tissue causes increased inflammation and intestinal permeability, and deletion of PD-L1 or treatment with anti-PD-L1 antibody attenuates sepsis-induced intestinal injury (27, 28).

### Therapeutic Targeting of the PD-1/PD-L1 Pathway

Antibodies blocking PD-1/PD-L1pathway are in clinical use for treating cancer (107), and remain of great interest as a means of treating sepsis. Hotchkiss and colleagues showed that administration of anti-PD-1 antibody at 24 h after CLP-induced sepsis induction, attenuated sepsis-induced T cell dysfunction and improved survival (108). Treatment with anti-PD-1 antibody after sepsis induction has also shown to improve T cell function, myeloid cell MHC-II expression and survival in a two-hit model of CLP-induced sepsis followed by fungal infection with *C. albicans* (38, 109). *Ex vivo* treatment with anti-PD-1 antibody reduces apoptosis and improves IFNγ production in circulating CD8 T cells obtained from septic patients (93). Likewise, Patera et al. showed that *ex vivo* treatment with anti-PD-1 antibody not only improves T cell function but also myeloid cell phagocytic function in peripheral blood leukocytes isolated from septic patients (25). Interestingly, a recent study by Phares et al. shows that treatment with a novel peptide (LD01) which blocks PD-1 signaling improved macrophage phagocytic capacity, T cell function and survival following CLP-induced sepsis (110). Collectively, these experimental studies show that treatment with anti-PD-1 is an attractive therapeutic option as it could be effective even when administered after sepsis induction. In a clinical trial, Hotchkiss and colleagues administered nivolumab, an anti-PD-1 antibody, to 31 septic patients beginning 24 h after sepsis diagnosis (111). Treatment did not result in significant induction of pro-inflammatory cytokine production, but adverse events possibly related to drug administration or immune dysfunction were identified in five subjects. Further large-scale trials are needed to assess the safety and efficacy of anti-PD-1 antibodies in septic patients.

Hotchkiss and colleagues recently conducted a Phase 1 clinical trial of anti-PD-L1 antibody (BMS-936559 from Bristol-Myers Squibb) in sepsis patients (111). Anti-PD-L1 was administered using a dose escalation strategy starting at 24 h after the onset of organ dysfunction to avoid treating during the peak inflammatory phase of sepsis. Importantly, treatment with anti-PD-L1 antibody was well tolerated and did not fuel inflammation in septic patients, and the antibody showed full PD-L1 receptor occupancy at 28 days after 900 mg single dose regimen (111). Previous studies by Patera et al. and Chang et al. demonstrated that *ex vivo* treatment with anti-PD-L1 antibody reverses sepsis-induced T cell dysfunction and improves phagocytic function of neutrophils and monocytes, in circulating blood cells obtained from septic patients (25, 93). In murine models of CLP-induced sepsis, treatment with anti-PD-L1 antibody reduces T cell apoptosis, enhances bacterial clearance, and reduces organ injury leading to improved survival (112, 113). Delayed treatment with anti-PD-L1 antibody at 24 h after induction of fungal sepsis with *C. albicans* also preserves T cell function and improves survival (38). Treatment with Compound 8, a novel PD-L1 blocking peptide, halved the mortality rate in a two-hit model of CLP-induced sepsis followed by fungal sepsis with *C. albicans* (114). Together, these studies suggest that targeting the PD-1/PD-L1 axis would likely be highly beneficial in the treatment of septic patients.

## 2B4

2B4 (CD244) is predominantly expressed on NK cells and T lymphocytes (31). The 2B4 belongs to the Signaling Lymphocyte Activation Molecule (SLAM) family of immunoregulatory receptors in the Ig superfamily (115). 2B4 receptor is composed of an extracellular Ig-like domains, a transmembrane region, and a cytoplasmic domain consisting of four ITSMs (115, 116). CD48, expressed on APCs, is the known ligand for 2B4 (32). High expression of 2B4 on T cells relays an inhibitory signal and low-moderate 2B4 expression leads to a co-stimulatory signal (116). Using a lymphocytic choriomeningitis virus (LCMV) infection model, Blackburn et al. showed that blocking the 2B4 pathway with CD48 antibody reduced IFNγ production in CD8 T cells expressing intermediate levels of 2B4, whereas increased IFNγ production in CD8 T cells expressing high levels of 2B4 (117). These studies demonstrate that high 2B4 expression leads to functional exhaustion of T cells (117). Interestingly, the signaling pathway upon CD48-2B4 ligation could be bidirectional. A study by Assarsson et al. showed that NK cells can augment T cell proliferation and activation, which involves interactions between 2B4 expressed on NK cells and CD48 expressed on CD8 T cells (118).

A seminal study by Chen al. showed significant upregulation of 2B4 on CD4 and CD8 T cells in human sepsis patients and in a CLP model of murine sepsis within 24 h of sepsis induction (33). Using conditional knock out mice, these studies revealed that deletion of 2B4 expressed specifically on CD4 T cells offers survival advantage following sepsis (33). Follow-up studies from the same research group showed 2B4 specifically mediated the loss of memory T cells, which was attenuated by 2B4 deletion leading to improved survival after sepsis (34). 2B4^+^ T cells from septic patients showed increased caspase-3/7^+^ apoptotic T cells, and 2B4 deletion reduced caspase-3/7^+^ apoptotic T cells in a murine CLP model of sepsis, thereby implicating 2B4 as an important mediator of sepsis-induced T cell apoptosis (34). Using a two-hit model of CLP sepsis followed by murine Epstein-Barr virus infection (gHV), a recent study by Xie et al. shows that deletion of 2B4 protects against sepsis-induced loss of antigen specific CD8 T cells and attenuates the increased viral load following sepsis (35). This study noted an important observation that CD8 T cells from wild type mice exhibited increased PD-1 expression, which was attenuated in 2B4 knockout mice, implying that 2B4 could modulate PD-1 expression during sepsis. These results suggest that blocking 2B4 pathway could serve to limit expression of multiple co-inhibitory receptors causing T cell exhaustion and open a new area of investigation for future studies. Therefore, 2B4 signaling pathway represents an attractive therapeutic target to reverse sepsis-induced T cell exhaustion and merits further investigation.

## Cytotoxic T Lymphocyte Antigen-4 (CTLA-4)

CTLA-4 (CD152) is a dimeric cell surface glycoprotein, which binds to CD80 and CD86 cell surface receptors on APCs (36). CTLA-4 is a known negative regulator of T cell effector functions and strongly counteracts CD28-mediated T cell co-simulation (42). CTLA-4 regulates T cell function through recruitment of cell intrinsic serine/threonine Protein Phosphatase 2A (PP2A), which inhibits CD3/CD28-mediated upregulation of cellular glucose metabolism *via* blocking of AKT phosphorylation (**Figure 1**) (81, 119). CTLA-4 signaling reduces cell surface expression of CD80 and CD86 on APCs *via* promoting their endocytosis, thereby limiting the availability of these co-stimulatory receptors for CD28 (119). CTLA-4 engagement reduces IL-2 receptor expression and IL-2 generation leading to cell cycle arrest, and reduced proliferation and activation of T cells (121, 122). Therefore, CTLA-4 signaling pathway downregulates T cell function *via* both cell intrinsic and extrinsic pathways, and represents an important immune checkpoint regulating T cell effector functions.

Limited studies have evaluated the role of CTLA-4 pathway during sepsis. Boomer et al. showed a time-dependent progressive increase in CTLA-4 expression on T cells in septic patients (123). Studies by Inoue et al. demonstrated that increased CTLA-4 expression on CD4 and CD8 T cells within 24 h of sepsis induction was associated with increased T cell depletion *via* apoptosis (37). Treatment with anti-CTLA4 antibody attenuates T cell apoptosis and improves survival in CLP induced sepsis (using a dose of 50 µg per mouse) and in a two-hit model of CLP induced sepsis followed by fungal infection with *C. albicans* (using a dose of 50 µg per mouse) (37, 38). A higher dose of anti-CTLA-4 antibody (200 µg) worsened survival outcomes, indicating that excess CTL-4 inhibition could fuel increased inflammation *via* uncontrolled T cell activation (37). Recent studies have also explored the correlation between single nucleotide polymorphisms (SNP) within the CTLA-4 gene and sepsis severity in humans. The functional SNPs such as rs231775, rs733618, and rs3087243 in the CTLA-4 gene have been shown to be associated with autoimmune diseases such as rheumatoid arthritis (124), Hashimoto thyroiditis (124, 125), and autoimmune diabetes (126, 127). These CTLA-4 SNPs are associated with higher T cell activation and proliferation capacity owing to a reduced CTLA-4 expression (128). Therefore, it is possible that the presence of specific CTLA-4 SNPs could be beneficial during sepsis in regard to alleviation of CTLA-4 mediated T cell exhaustion. Interestingly, a recent study by Mewes et al. reported a significantly lower 28- and 90-day mortality risk among septic Caucasian patients with rs231775 GG homozygous SNP in CTLA-4 gene (129). A follow-up study from the same group further demonstrated that CTLA-4 rs3087243 G allele carriers showed lower 28- and 90-day mortality risk (130). These demonstrate that specific SNPs in CTLA-4 could serve as prognostic predictors in septic patients. However, the significance of these specific CTLA-4 SNPs in regulating T cell function during sepsis remains to be explored, and hold significant potential for future research in personalized medicine approach.

## B and T Lymphocyte Attenuator (BTLA) and Herpes Virus Entry Mediator (HVEM)

BTLA (CD272) is a type I transmembrane glycoprotein, and its cytoplasmic domain is comprised of distinct intracellular motifs including growth factor receptor-bound protein-2 (Grb-2), ITIM and ITSM (131). BTLA exhibits bidirectional regulation, as the ITIM motif could deliver inhibitory signals *via* SHP1 and SHP2 mediated inhibition of cell activation, and stimulatory signals *via* Grb-2 motif which recognizes Grb-2 protein leading to activation of the PI3K pathway (132, 133). BTLA is expressed on T cells and innate immune cells including monocytes, macrophages, and dendritic cells (41, 42). BTLA interacts with HVEM (CD270 or TNFRSF14), a type I transmembrane receptor belonging to the tumor necrosis factor receptor superfamily, and is known to also interact with multiple additional ligands such as CD160, LIGHT, and LTα (39, 134). Montgomery et al. discovered HVEM as a required receptor for cellular entry of herpes simplex virus-1 (135). HVEM has diverse cell type distribution on APCs such as monocytes, dendritic cells, neutrophils, and NK cells, and it is also expressed on resting T cells and immature B cells (39, 40). Engagement of BTLA with its receptor HVEM predominantly inhibits TCR signaling mediated effector functions including T cell activation and proliferation (133).

Recent studies have implicated a role for BTLA as an important immune checkpoint molecule during sepsis-induced immunosuppression. In some of the first evidence supporting this role, Boomer et al. demonstrated the increased presence of HVEM in postmortem lungs of septic patients as compared to non-septic controls (23). Shubin et al. showed that increased BTLA expression on CD4 T cells is predictive of susceptibility to secondary nosocomial infections among sepsis patients and ablation of BTLA protects against sepsis-induced CD4 T cell depletion in a murine model of CLP-induced sepsis (43). In contrast to these findings, Spec et al. observed no difference between leukocyte BTLA expression in septic patients infected by fungal *Candida albicans* infection (96), which could point to a difference in the role of BTLA depending on the etiology of sepsis. Shubin and colleagues also demonstrated that sepsis leads to increased infiltration of high BTLA and HVEM expressing innate immune cells including macrophages, inflammatory monocytes, dendritic cells, and neutrophils at the site of infection in the peritoneum (136). Using BTLA knockout mice, this study also showed enhanced activation of innate leukocytes, reduced bacterial burden, and attenuation of organ injury leading to improved survival (136). Therefore, targeting BTLA could serve to activate both innate and adaptive immunity during sepsis. Higher plasma levels of soluble BTLA (sBTLA) greater than 21 ng/ml, were predictive of five-fold increase in mortality among sepsis patients, implying sBTLA as a prognostic marker during sepsis (137). However, the functional significance of increased sBTLA during sepsis is unknown. Treatment with anti-BTLA antibody in a two-hit murine model of hemorrhage followed by sepsis lead to increased inflammation, organ injury and mortality (138). Therefore, future studies employing anti-BTLA antibody should be carefully evaluated in a dose and time response manner along with its effects on T cell and myeloid functions.

## Lymphocyte Activation-Gene-3 (LAG-3) and T Cell Membrane Protein-3 (TIM-3)

LAG-3 and TIM-3 are the lesser investigated immune checkpoints during sepsis as compared to those discussed above. LAG-3 (CD223) is expressed on T cells and belongs to the Ig superfamily. It binds to major histocompatibility class II (MHC II) on APCs and competes for binding of the MHC molecules to TCR on T cells, thereby inhibiting T cell immune responses (139). LAG-3 signaling is mediated *via* its intracellular KIEELE motif, which causes cell cycle arrest in T cells and suppresses T cell proliferation (140). TIM-3 is a member of the Ig superfamily and it interacts with CEACAM1 (carcinoembryonic antigen-related cell adhesion molecule 1) or Galectin 9 (42), both of which are expressed on a variety of immune cells and tissues (45, 48, 49). TIM-3/Galectin 9 interaction has been shown to cause apoptosis in CD4 T cells (141).

Using a CLP model of murine sepsis, a recent study by Lou et al. showed increased LAG-3 expression not only on CD4 and CD8 T cells, but also on B cells, regulatory T cells, and dendritic cells, and treatment with anti-LAG-3 antibody improved T cell function, bacterial clearance and survival after sepsis (44). Importantly, anti-LAG-3 antibody was administered in a therapeutic manner at 3 h after sepsis induction, which increases the clinical relevance. A recent study by Niu et al. shows T cells co-expressing LAG-3 and PD-1 synergistically inhibit CD4 and CD8 T cells, and this correlated with increased length of hospital stay and higher mortality (142). Interestingly, this study showed that LAG-3 was only significantly elevated at day 5 post sepsis, as compared to PD-1 which was elevated early at 12 h after sepsis, indicating an important role for LAG-3 immunosuppression during progression of sepsis. Studies by Boomer et al. showed increased expression of LAG-3 and TIM-3 on CD4 T cells, with a relatively higher level of LAG-3 on CD8 T cells as compared to TIM-3 (122). Immune checkpoints are critical in maintaining immune tolerance and homeostasis to limit excess inflammation. Studies by Yang et al. demonstrated a direct correlation between reduced TIM-3 mRNA expression in peripheral blood mononuclear cells and increased pro-inflammatory status, and blocking TIM-3 signaling with anti-TIM-3 antibody worsened outcomes in murine model of CLP-induced sepsis (47). In line with these findings, the same research group demonstrated that soluble TIM-3 immunoglobulin which blocks TIM-3 signaling exacerbates inflammation and T cell apoptosis during sepsis, which was attenuated in mice overexpressing TIM-3 (46). Studies by Ren et al. show increased TIM-3 expression on monocytes in sepsis patients, but no change in severe sepsis or septic shock patients (143). Furthermore, soluble TIM-3 levels correlated with mortality in the septic shock group alone (143). Spec et al. showed no difference in TIM-3 expression on CD4 and CD8 T cell in sepsis patients affected by *C. albicans* infection, implying that fungal etiology of sepsis may not involve TIM-3 (95). It is important to note that studies showing increased inflammation with anti-TIM-3 administered it in a prophylactic manner at least 24 h before sepsis induction (46, 47). Based on all these findings, it is possible that TIM-3 serves to limit excess inflammation during sepsis and strategies to prophylactically block TIM-3 might accentuate inflammation during sepsis. Future studies should carefully evaluate a dose and time dependent response of anti-TIM-3 antibody as a therapeutic during sepsis, and test whether blocking TIM-3 signaling during the acute inflammatory phase after sepsis induction is a viable strategy.

## Immune Checkpoint Inhibitors as a Therapeutic Strategy to Protect Against Nosocomial Infections

Although judicious fluid resuscitation and antibiotic strategies have decreased short-term death among sepsis patients, sepsis survivors manifest increased morbidity and mortality from secondary nosocomial infections. Sepsis-induced immunosuppression increases susceptibility to secondary life-threatening infections caused by microbes such as *Pseudomonas, Candida, Acinetobacter, and Enterococcus* (15). Septic patients also demonstrate increased reactivation of latent viruses including cytomegalovirus and herpes simplex (144, 145). As discussed above, increased expression of immune checkpoints including PD-1, PD-L1, 2B4, and BTLA play a major role in sepsis-induced immunosuppression. Targeting immune checkpoints could be harnessed to reinvigorate host immune responses and protect critically ill patients against secondary hospital acquired infections. Blocking PD-1/PD-L signaling pathways is protective in two-hit models of CLP-induced bacterial sepsis followed by fungal *C. albicans* infection (38, 108, 113). Double LAG-3 and PD-1 positive T cells show significant dysfunction and LAG-3 is upregulated later in the course of sepsis progression in patients (142). 2B4 is implicated in inhibiting the T cell specific responses, thereby compromising host immune responses (34, 35). Apart from sepsis, critically ill patients from other etiologies could also be a target population which could benefit from targeting immune checkpoints. Patients with acute liver failure demonstrate reduced proliferation capacity and increased expression of CTLA-4 on circulating CD4 T cells, and *ex vivo* treatment with anti-CTLA-4 antibody restores the proliferative response (146). Studies by Shubin et al. demonstrated increased risk of acquiring nosocomial infections among non-septic critically ill patients with greater than 80% BTLA expression on CD4 T cells (43). Therefore, this evidence brings to light an important observation that therapeutics targeting immune checkpoints could be prime candidates to limit the risk of developing secondary infections among septic patients, and this merits detailed future investigations using clinically relevant models of sepsis.

## Can Therapeutics Targeting Immune Checkpoints Augment Trained Immunity-Mediated Protection Against Infections?

Trained immunity refers to a long-term functional reprogramming of innate leukocytes, evoked by a prior exposure to microbial ligands or a non-lethal infectious insult, which leads to an amplified anti-microbial response to a broad range of secondary infections (147). Trained immunity induced by microbial ligands such as Toll-like receptor 4 agonists (TLR4), including Monophosphoryl lipid-A (MPLA) and fungal ligand β-glucan, potently protects against life-threatening pathogens such as *Pseudomonas aeruginosa*, *Staphylococcus aureus*, and *Mycobacterium tuberculosis* (148–151). TLR4 agonist-induced trained immunity involves metabolic reprogramming of macrophages including mitochondrial biogenesis and augmentation of glycolysis and mitochondrial oxidative phosphorylation (149, 152).

The general consensus holds that trained immunity is predominantly mediated *via* innate immune cells and independent of the adaptive immunity (147, 149). As discussed above, immune checkpoints, specifically the PD-1/PD-L pathway also affects innate leukocyte responses during sepsis (20). Studies by Huang et al. showed increased PD-1 expression on macrophages at the site of infection correlated with their dysfunction and while deletion of PD-1 protected against sepsis lethality (153). PD-L1 delivers a constitutive inhibitory signal within macrophages, and treatment with anti-PD-L1 antibody activates both murine and human macrophages (89). Stimulation of TLR4 receptor by lipopolysaccharide and MPLA has been shown to induce PD-L1 expression on innate leukocytes including macrophages and dendritic cells (154, 155). Metabolic reprogramming is the hallmark of trained immunity, and interaction between immune checkpoint pathways and cellular metabolism in innate leukocytes during sepsis is not well characterized. A recent study by Deng et al. shows that pyruvate kinase M2 (glycolytic enzyme) and STAT1 signaling drive upregulation of PD-L1 expression on macrophages (100). Hypoxia-inducible factor-1α (HIF-1α) signaling has also been shown to upregulate PD-L1 expression in endotoxin tolerant monocytes isolated from septic patients, and *ex vivo* co-culture experiments using knockdown of HIF-1α or PD-L1 from septic monocytes significantly improved T cell proliferation capacity (156). Interestingly, induction of trained immunity *via* ligands such as β-glucan and MPLA require HIF-1α mediated increase in glycolysis (157, 158). Therefore, it is possible that stimulation with ligands which induce trained immunity such as MPLA could also cause increased PD-L1 expression, and the therapeutic efficacy of these ligands could be further boosted by blocking specific immune checkpoint pathways such as PD-1/PD-L. Immune checkpoint blockers could thus serve as adjunct therapeutics in combination with clinically applicable microbial ligands such as MPLA. However, the role of immune checkpoints in regulating leukocyte metabolic reprogramming and generation of microbial ligand-induced trained immunity, and the metabolic effects of blocking immune checkpoint pathways during sepsis are currently unknown. Apart from innate leukocytes, signaling through immune checkpoints including PD-1 and CTLA-4 predominantly impair the uptake of glucose and subsequent glucose metabolism *via* inhibiting glycolysis, leading to impaired T cell effector responses (80, 159). PD-1 signaling impairs T cell functions by inhibiting PI3K and Akt signaling, which diverts cellular metabolism away from glycolysis to fatty acid oxidation (159). CTLA-4 also blocks the Akt pathway and glycolysis, although through a different mechanism involving PP2A (80, 118). Therefore, the T cell protective effects of therapeutics blocking immune checkpoints such as PD-1 and CTLA-4 pathways during sepsis could also involve metabolic reprogramming and re-invigoration of the glycolytic pathway, which will be an interesting topic for future studies.

## Concluding Remarks for Translational Application of Therapeutics Targeting Immune Checkpoints

Therapeutics targeting immune checkpoints hold significant potential to reverse sepsis-induced immunosuppression and preserve host immunity against primary and secondary infections. Future studies should also evaluate a combinatorial therapeutic approach using immune checkpoint blockade in conjunction with other therapeutics such as interleukin-7, which augments T cell functions, as well as microbial ligands such as MPLA which induce trained immunity mediated augmentation of innate immune functions. A recent study by Hotchkiss and colleagues demonstrated the safety of PD-L1 antibody in septic patients (110). However, cancer clinical trials have documented a broad spectrum of serious adverse events associated with immune checkpoint blockade such as liver injury, thyroiditis, colitis, pneumonitis, thrombocytopenia, vasculitis and others (160), and in a murine model of CLP sepsis, anti-CTLA-4 antibody worsened survival at higher doses (37). Clinical use of such therapeutics will need to be tailored to individual patients based on immunophenotypic analysis of circulating immune cells. The concurrent existence of proinflammatory and immunosuppressed states in sepsis calls for a careful consideration of the translational application of immune checkpoint targeted therapeutics. Nonetheless, many studies reviewed here show therapeutic potential for this class of drugs in a disease currently managed only with source control and supportive care.

## Author Contributions

NKP and ERS conceptualized the manuscript. NKP, ERS, MM, and TKP contributed to writing the initial draft of the manuscript. MM drafted the table. MM and TKP drafted the figure. ERS, JKB, and AH critically revised the manuscript for important intellectual concepts. NKP supervised the drafting and performed final editing. All authors contributed to the article and approved the submitted version.

## Funding

This work was supported by National Institutes of Health (NIH) grants R01 GM119197 and R01 AI151210 to ERS; T32 GM108554-05, Shock Society Faculty Research Award and Vanderbilt Faculty Scholars Award to NKP, R01 GM12171 to JKB, K08 GM123345 to AH, and T32 GM007347-41 to MM.

## Conflict of Interest

The authors declare that the research was conducted in the absence of any commercial or financial relationships that could be construed as a potential conflict of interest.
